# Co-Circulation of the Rare CPV-2c with Unique Gln370Arg Substitution, New CPV-2b with Unique Thr440Ala Substitution, and New CPV-2a with High Prevalence and Variation in Heilongjiang Province, Northeast China

**DOI:** 10.1371/journal.pone.0137288

**Published:** 2015-09-08

**Authors:** Yufei Geng, Donghua Guo, Chunqiu Li, Enyu Wang, Shan Wei, Zhihui Wang, Shuang Yao, Xiwen Zhao, Mingjun Su, Xinyu Wang, Jianfa Wang, Rui Wu, Li Feng, Dongbo Sun

**Affiliations:** 1 College of Animal Science and Veterinary Medicine, Heilongjiang Bayi Agricultural University, No. 2 Xinyang Road, Sartu District, Daqing 163319, P. R. China; 2 National Key Laboratory of Veterinary Biotechnology, Harbin Veterinary Research Institute of the Chinese Academy of Agricultural Sciences, No. 427 Maduan Street, Nangang District, Harbin 150001, P. R. China; Fudan University, CHINA

## Abstract

To trace evolution of canine parvovirus-2 (CPV-2), a total of 201 stool samples were collected from dogs with diarrhea in Heilongjiang province of northeast China from May 2014 to April 2015. The presence of CPV-2 in the samples was determined by PCR amplification of the VP2 gene (568 bp) of CPV-2. The results revealed that 95 samples (47.26%) were positive for CPV-2, and they showed 98.8%–100% nucleotide identity and 97.6%–100% amino acid identity. Of 95 CPV-2-positive samples, types new2a (Ser297Ala), new2b (Ser297Ala), and 2c accounted for 64.21%, 21.05%, and 14.74%, respectively. The positive rate of CPV-2 and the distribution of the new2a, new2b and 2c types exhibited differences among regions, seasons, and ages. Immunized dogs accounted for 48.42% of 95 CPV-2-positive samples. Coinfections with canine coronavirus, canine kobuvirus, and canine bocavirus were identified. Phylogenetic analysis revealed that the identified new2a, new2b, and CPV-2c strains in our study exhibited a close relationship with most of the CPV-2 strains from China; type new2a strains exhibited high variability, forming three subgroups; type new2b and CPV-2c strains formed one group with reference strains from China. Of 95 CPV-2 strains, Tyr324Ile and Thr440Ala substitutions accounted for 100% and 64.21%, respectively; all type new2b strains exhibited the Thr440Ala substitution, while the unique Gln370Arg substitution was found in all type 2c strains. Recombination analysis using entire VP2 gene indicated possible recombination events between the identified CPV-2 strains and reference strains from China. Our data revealed the co-circulation of new CPV-2a, new CPV-2b, and rare CPV-2c, as well as potential recombination events among Chinese CPV-2 strains.

## Introduction

Canine parvovirus-2 (CPV-2) causes a highly contagious and often fatal disease characterized by vomiting and hemorrhagic gastroenteritis in dogs of all ages, and by myocarditis in pups of less than three months of age [1]. The vaccine could provide protective immunity against the classic CPV-2 infection in canine populations, reducing the mortality of animals and spreading of the virus. However, the long-term interaction of the virus and its host may have led to the emergence of CPV-2 antigenic variants. During the past decades, new antigenic types of the CPV-2a, CPV-2b, CPV-2c, new CPV-2a, and new CPV-2b were frequently reported in the dog population, and they replaced the original CPV-2, which has caused widespread concern [2–9]. Therefore, molecular epidemiological investigation of CPV-2 is an undergoing hotspot issue worldwide.

The VP2 protein of CPV-2 is the major capsid protein, and it is highly antigenic, playing important roles in determining viral host ranges and tissue tropisms [10, 11]. At present, VP2 is widely used for the identification and monitoring of the types of CPV-2 circulating in the canine population based on the amino acid residues of the VP2 protein at positions ^426^Asp/Glu/Asn and ^297^Ser/Ala [10, 12–15]. In the current study, the molecular epidemiology of the CPV-2 strains circulating in northeast China was investigated by polymerase chain reaction (PCR) targeting the partial VP2 gene. Moreover, potential recombination events of the identified CPV-2 strains were analyzed using the selected entire VP2 gene. Our aim was to provide insights into the epidemiology and genetic diversity of the CPV-2 strains circulating in northeast China, which can be useful for preventing and controlling CPV-2 infections.

## Materials and Methods

### Ethics statement

All animal experiments were conducted according to the Guide for the Care and Use of Laboratory Animals of Harbin Veterinary Research Institute (HVRI), Chinese Academy of Agricultural Sciences, China. Sampling and data publication also were approved by animal’s owners. The field study did not involve endangered or protected species. No specific permissions were required for locations of samples because the samples were collected from public areas or non-protection areas.

### Sampling

Commercial virus sampling tubes (YOCON Biological Technology Co. Ltd., Beijing, China) containing 3.5 mL of Hank’s balanced salt solution, streptomycin, and penicillin, were used to collect fecal samples. A total of 201 fecal samples were collected in the form of rectal swabs from animal hospitals of the Harbin, Daqing, and Mudanjiang districts of Heilongjiang province in northeast China from May 2014 to April 2015. These animals, which were aged between 1 month and 1 year, included animals with symptoms of different degrees of diarrhea, animals with or without a vaccination history, and animals from different breeds and both genders. Of the 201 samples, 141 were collected in Harbin, 20 were collected in Daqing, and 40 were collected in Mudanjiang. All rectal swab samples were stored at −80°C, and they were also used for etiological investigations in our other studies.

### DNA extraction

After 1 mL of fecal samples was centrifuged at 1,500 × *g* for 10 min at 4°C, the supernatant of each sample was transferred to a 1.5 mL EP tube. Genomic DNA was extracted from the supernatant using a commercial TIANamp Stool DNA Kit (Tiangen Biotech Co., Ltd., Beijing, China) according to the manufacturer’s instructions. The extracted genomic DNAs were stored at −40°C. A commercial vaccine was used as a positive control, and distilled water was used as a negative control.

### Sequencing of the VP2 fragment

For all samples, 568 bp of the VP2 gene was amplified using the primers CPV-FP (5’−TGATTGTAAACCATGTAGACTAAC−3’) and CPV-RP (5’−TAATGCAGTTAAAGGACCATAAG−3’) as described by Mittal et al. (2014). PCR amplifications were conducted in a 50 μL reaction volume containing 0.1 μM of forward primer, 0.1 μM of reverse primer, 4 μL of genomic DNA, 25μL of EmeraldAmp PCR Master Mix (2 × Premix) (TaKaRa Biotechnology Co. Ltd., Dalian, China), and an appropriate volume of double-distilled (dd) H_2_O. The mixtures were amplified by 35 cycles of 94°C for 30 s, 55°C for 30 s, and 72°C for 1 min, with a final extension at 72°C for 10 min using an automated Applied Biosystems GeneAmp PCR System 9700 thermal cycler (Thermo Fisher Scientific, Waltham, MA, USA). After the amplification, products were purified using the AxyPrep DNA Gel Extraction kit (A Corning Brand, Suzhou, China), and they were directly subjected to Sanger sequencing. Each sample was sequenced three times. Searches for nucleotide similarities were conducted using the BLAST algorithm at the National Center for Biotechnology Information (NCBI) (http://www.ncbi.nlm.nih.gov/blast). Sequence analysis was performed using the EditSeq program in the Lasergene DNASTAR version 5.06 software (DNASTAR Inc., Madison, WI, USA). Multiple sequence alignments were performed using the Multiple Sequence Alignment program of the DNAMAN version 6.0 software (Lynnon BioSoft, Point-Claire, Quebec, Canada). A total of 95 sequences (507 bp in length) were submitted to the NCBI Genbank (http://www.ncbi.nlm.nih.gov) under the accession numbers KT074258–KT074352.

### Phylogenetic analysis

For the phylogenetic analysis, the VP2 gene sequences of CPV-2, CPV-2a, CPV-2b, CPV-2c, new CPV-2a, and new CPV-2b strains from different geographical locations within China and the rest of the world were retrieved from the NCBI nucleotide database. A nucleotide phylogenetic tree of the VP2 fragments (507 bp) of CPV-2 was generated from the ClustalX-generated alignments by MEGA6.06 software using the neighbor-joining method [16]. Neighbor-joining phylogenetic tree was built with the *p*-distance model and 1000 replicates of interior-branch test; otherwise, the default parameters in MEGA 6 were used. The support of reliability (above 50%) was shown in the obtained phylogenetic tree.

### Sequencing of the entire selected VP2 genes

A total of 13 entire VP2 genes identified in our study, representing different genetic diversity in the phylogenetic tree of the 507 bp VP2 fragments of CPV-2, were chosen for recombination analysis. The entire VP2 gene of CPV-2 was amplified using the primers CPVvp2-F (5'-AACTAAAAGAAGTAAACCAC-3') and CPVvp2-R (5'-TGTAATAAACATAAAAACAT-3'), which resulted in a 1882 bp fragment covering the open reading frame of the VP2 gene. PCR amplifications were conducted in a 50 μL reaction volume containing 0.1 μM of forward primer, 0.1 μM of reverse primer, 4 μL of genomic DNA, 5 μL of 10 × buffer, 0.5 U of ExTaq DNA polymerase (TaKaRa), and an appropriate volume of ddH_2_O. The mixtures were amplified by 35 cycles of 94°C for 1 min, 50°C for 1 min, and 72°C for 2 min, with a final extension at 72°C for 10 min using an automated Applied Biosystems GeneAmp PCR System 9700 thermal cycler. The VP2 PCR products were purified using the AxyPrep DNA Gel Extraction kit (Corning), and then purified products were directly subjected to Sanger sequencing. Each sample was sequenced three times. All sequences were submitted to the NCBI Genbank (http://www.ncbi.nlm.nih.gov) under the accession numbers KT156825–KT156837.

### Recombination analysis

Thirteen entire VP2 sequences in this study and 25 VP2 gene reference sequences from different areas in China were used for the recombination analysis (Table 1). The 38 VP2 genes were aligned in the ClustalX (1.83) program, and then were screened for possible recombination by using the Recombination Detection Program (RDP) [17], GENECONV [18], BOOTSCAN [19], MaxChi [20], CHIMAERA [21], and SISCAN [22] methods embedded in RDP4. Only potential recombination events detected by two or more of the programs, coupled with phylogenetic evidence of recombination, were considered significant using the highest acceptable *P*-value cutoff of 0.05.

**Table 1:** List of the entire VP2 gene for recombination analysis.

No.	Strains	Accession no.	Genotyping	Location in China	Year
1	MDJ-1	KT156825	New2a	Northeast (Mudanjiang)	2014
2	MDJ-7	KT156826	New2a	Northeast (Mudanjiang)	2014
3	MDJ-9	KT156827	New2a	Northeast (Mudanjiang)	2014
4	MDJ-15	KT156828	New2a	Northeast (Mudanjiang)	2014
5	MDJ-20	KT156829	New2a	Northeast (Mudanjiang)	2015
6	MDJ-22	KT156830	New2a	Northeast (Mudanjiang)	2015
7	HRB-A4	KT156831	New2b	Northeast (Harbin)	2014
8	HRB-A6	KT156832	New2c	Northeast (Harbin)	2014
9	HRB-b2	KT156833	New2b	Northeast (Harbin)	2014
10	HRB-e2	KT156834	New2a	Northeast (Harbin)	2015
11	HRB-ee7	KT156835	New2a	Northeast (Harbin)	2014
12	HRB-F8	KT156836	New2a	Northeast (Harbin)	2014
13	HRB-J8	KT156837	New2b	Northeast (Harbin)	2014
14	bj-5	GQ169549	New2a	North (Beijing)	2010
15	Wu	KJ674820	New2a	South-central (Wuhan)	2013
16	CPV/WH02/06	EU377537	New2a	South-central (Wuhan)	2006
17	06–11-NJ	FJ432716	New2a	East (Nanjing)	2006
18	08–5-WH	FJ432717	New2a	South-central (Wuhan)	2008
19	Wh-3	GQ169539	New2a	South-central (Wuhan)	2009
20	1-nj	GQ169550	New2a	East (Nanjing)	2009
21	09/09	GU452713	New2a	NA	2009
22	11/09	GU452715	New2a	NA	2009
23	CPV-6(10)	JQ743904	New2a	NA	2010
24	CPVSH-3/2011	JX121627	New2a	East (Shanghai)	2011
25	GZ0202	GU569937	New2b	Southwest (Guizhou)	2002
26	YN0201	GU569938	New2b	Southwest (Yunnan)	2002
27	YN0203	GU569940	New2b	Southwest (Yunnan)	2002
28	GZ0201	GU569944	New2b	Southwest (Guizhou)	2002
29	CPV-4(10)	JQ743890	New2b	NA	2010
30	CPV-10(10)	JQ743891	New2b	NA	2010
31	CPV-BG(11)	JQ743892	New2b	NA	2011
32	CPV-HS(11)	JQ743893	New2b	NA	2011
33	CPV-BM(11)	JQ743894	New2b	NA	2011
34	CPV-2b-wuhan2	KC881278	New2b	South-central (Wuhan)	2010
35	06/09	GU380303	2c	NA	2009
36	08/09	GU380305	2c	NA	2009
37	G1	KF482468	2c	South	2009
38	G15	KF482471	2c	South	2009

### Screening for canine enteric pathogens

Fecal samples that tested positive for CPV-2 were also screened for canine bocavirus (CBoV), canine coronavirus (CCoV), canine astrovirus (CaAstV), canine norovirus (CNoV), canine kobuvirus (CaKV), and group A-rotavirus (RV-A), by either PCR or reverse transcription-PCR (RT-PCR) and sequencing, as previously described [23–28].

## Results

### Investigation and genotyping of CPV-2

A total of 201 samples from the Harbin, Daqing, and Mudanjiang districts of Heilongjiang province in northeast China were detected via PCR amplification of 568 bp of the VP2 gene of CPV-2. Nucleotide sequences of the partial VP2 of CPV-2 strains identified in our study were shown in Supporting Information (S1 Fig). The characteristics of the CPV-2-positive dogs, the genotypes of CPV-2 strains, and the amino acid substitutions of the VP2 protein are shown in Table 2, and a further analysis of the CPV-2-positive samples is shown in Table 3. Of the 201 samples, 47.26% (95/201) were positive for CPV-2, and three types, new2a (Ser297Ala), new2b (Ser297Ala), and 2c, were found, accounting for 64.21%, 21.05%, and 14.74%, respectively. The new2a type was identified in Harbin, Daqing, and Mudanjiang; the new2b type was identified in Harbin and Daqing; and the 2c type was identified in Harbin and Mudanjiang.

**Table 2:** Characteristics of the CPV-2 positive dogs, the genotypes of CPV-2 strains, and amino acid substitution of the VP2 protein in northeast China.

	No.	Strain	Accession No.	Collection date	Location	Breed	Gender	Age	Vaccine	Other enteric viruses (Accession No.)	CPV type	Substitution of amino acid residues in VP2 protein of CPV					
												297	324	370	419	426	440
**Reference strains**	1	CPV-b	M38245	1996	USA	NA	NA	NA	NA	NA	2	Ser	Tyr	Gln	Asn	Asn	Thr
	2	YB8301	GU569943	1983	China	NA	NA	NA	NA	NA	2	Ser	Try	Gln	Asn	Asn	Thr
	3	CPV-15	M24003	1984	USA	NA	NA	NA	NA	NA	2a	Ser	Tyr	Gln	Asn	Asn	Thr
	4	CC8601	GU569948	1986	China	NA	NA	NA	NA	NA	2a	Ser	Tyr	Gln	Asn	Asn	Thr
	5	CPV-39	M74849	1984	USA	NA	NA	NA	NA	NA	2b	Ser	Tyr	Gln	Asn	Asp	Thr
	6	56/00	FJ222821	2009	Italy	NA	NA	NA	NA	NA	2c	Ala	Tyr	Gln	Asn	Glu	Thr
	7	06/09	GU380303	2009	China	NA	NA	NA	NA	NA	2c	Ala	Ile	Gln	Asn	Glu	Thr
	8	G1	KF482468	2009	China	NA	NA	NA	NA	NA	2c	Ala	Tyr	Gln	Asn	Glu	Thr
	9	CPV-435	AY742953	2005	USA	NA	NA	NA	NA	NA	New2a	Ala	Tyr	Gln	Asn	Asn	Thr
	10	Wh-6	GQ169542	2009	China	NA	NA	NA	NA	NA	New2a	Ala	Ile	Gln	Asn	Asn	Thr
	11	CPVSH-2/2011	JX121626	2011	China	NA	NA	NA	NA	NA	New2a	Ala	Ile	Gln	Asn	Asn	Ala
	12	CPV-436	AY742955	2005	USA	NA	NA	NA	NA	NA	New2b	Ala	Tyr	Gln	Asn	Asp	Thr
	13	GZ0201	GU569944	2002	China	NA	NA	NA	NA	NA	New2b	Ala	Tyr	Gln	Asn	Asp	Thr
	14	CPV-2b-wuhan2	KC881278	2010	China	NA	NA	NA	NA	NA	New2b	Ala	Ile	Gln	Asn	Asp	Ala
**Strains in this study**	1	MDJ-1	KT074258	Dec-2014	MDJ	SH	F	2M	Yes	CCoV (KT192642)	New2a	Ala	Ile	Gln	Asn	Asn	Ala
	2	MDJ-22	KT074265	Oct-2014	MDJ	GM	F	6M	Yes	—	New2a	Ala	Ile	Gln	Asn	Asn	Ala
	3	MDJ-28	KT074267	Dec-2014	MDJ	JS	M	3M	Yes	—	New2a	Ala	Ile	Gln	Asn	Asn	Ala
	4	MDJ-29	KT074268	May-2015	MDJ	MX	M	5M	No	—	New2a	Ala	Ile	Gln	Asn	Asn	Ala
	5	MDJ-32	KT074269	Jan-2015	MDJ	NA	NA	NA	Yes	—	New2a	Ala	Ile	Gln	Asn	Asn	Ala
	6	MDJ-40	KT074272	May-2015	MDJ	PD	F	NA	Yes	—	New2a	Ala	Ile	Gln	Asn	Asn	Ala
	7	DQ-alpha1	KT074273	Dec-2014	DQ	NA	F	3M	No	CCoV (KT192660)	New2a	Ala	Ile	Gln	Asn	Asn	Ala
	8	DQ-beta1	KT074274	May-2015	DQ	PD	NA	3M	Yes	—	New2a	Ala	Ile	Gln	Asn	Asn	Ala
	9	HRB-A5	KT074278	Sep-2014	HRB	MX	M	NA	NA	—	New2a	Ala	Ile	Gln	Asn	Asn	Ala
	10	HRB-A8	KT074280	Oct-2014	HRB	BF	M	4M	NA	CCoV (KT192655), CaKV (KT210398)	New2a	Ala	Ile	Gln	Asn	Asn	Ala
	11	HRB-B0	KT074279	Oct-2014	HRB	PM	F	3M	Yes	CCoV (KT192665), CaKV (KT210404)	New2a	Ala	Ile	Gln	Asn	Asn	Ala
	12	HRB-B7	KT074283	Oct-2014	HRB	MI	F	3M	Yes	CCoV (KT192668)	New2a	Ala	Ile	Gln	Asn	Asn	Ala
	13	HRB-b9	KT074318	Sep-2014	HRB	NA	M	3M	Yes	—	New2a	Ala	Ile	Gln	Asn	Asn	Ala
	14	HRB-C2	KT074285	Oct-2014	HRB	TM	F	NA	No	CCoV (KT192678), CaKV (KT210410)	New2a	Ala	Ile	Gln	Asn	Asn	Ala
	15	HRB-C5	KT074286	Oct-2014	HRB	YT	M	NA	No	—	New2a	Ala	Ile	Gln	Asn	Asn	Ala
	16	HRB-C6	KT074287	Oct-2014	HRB	BF	F	4M	Yes	—	New2a	Ala	Ile	Gln	Asn	Asn	Ala
	17	HRB-C9	KT074289	Oct-2014	HRB	MX	F	3M	Yes	—	New2a	Ala	Ile	Gln	Asn	Asn	Ala
	18	HRB-b5	KT074290	Oct-2014	HRB	NA	NA	3M	No	—	New2a	Ala	Ile	Gln	Asn	Asn	Ala
	19	HRB-bb7	KT074281	Oct-2014	HRB	GM	NA	2M	NA	CCoV (KT192671),	New2a	Ala	Ile	Gln	Asn	Asn	Ala
	20	HRB-bb8	KT074284	Oct-2014	HRB	SM	F	2M	No	CCoV (KT192672), CaKV (KT210406)	New2a	Ala	Ile	Gln	Asn	Asn	Ala
	21	HRB-dd2	KT074291	Nov-2014	HRB	BD	M	5M	NA	—	New2a	Ala	Ile	Gln	Asn	Asn	Ala
	22	HRB-D4	KT074294	Oct-2014	HRB	YT	F	4M	NA	CCoV (KT192683)	New2a	Ala	Ile	Gln	Asn	Asn	Ala
	23	HRB-D8	KT074295	Nov-2014	HRB	YT	M	3.5M	No	—	New2a	Ala	Ile	Gln	Asn	Asn	Ala
	24	HRB-D9	KT074296	Nov-2014	HRB	NA	NA	3.5M	Yes	CaKV (KT210414)	New2a	Ala	Ile	Gln	Asn	Asn	Ala
	25	HRB-E0	KT074297	Nov-2014	HRB	NA	F	3.5M	No	—	New2a	Ala	Ile	Gln	Asn	Asn	Ala
	26	HRB-E1	KT074298	Nov-2014	HRB	NA	M	3.5M	Yes	CCoV (KT192686)	New2a	Ala	Ile	Gln	Asn	Asn	Ala
	27	HRB-e2	KT074299	Apr-2015	HRB	NA	M	3M	No		New2a	Ala	Ile	Gln	Asn	Asn	Ala
	28	HRB-E8	KT074300	Nov-2014	HRB	GM	M	3M	Yes	CCoV (KT192687), CaKV (KT210415)	New2a	Ala	Ile	Gln	Asn	Asn	Ala
	29	HRB-F0	KT074301	Feb-2015	HRB	SM	F	4M	Yes	—	New2a	Ala	Ile	Gln	Asn	Asn	Ala
	30	HRB-F4	KT074302	Nov-2014	HRB	NA	M	NA	No	—	New2a	Ala	Ile	Gln	Asn	Asn	Ala
	31	HRB-G4	KT074305	Mar-2015	HRB	YT	F	4M	Yes	—	New2a	Ala	Ile	Gln	Asn	Asn	Ala
	32	HRB-H2	KT074307	Dec-2014	HRB	PD	F	3M	NA	CaKV (KT210423)	New2a	Ala	Ile	Gln	Asn	Asn	Ala
	33	HRB-I1	KT074308	Sep-2014	HRB	NA	F	4M	NA	CCoV (KT192697)	New2a	Ala	Ile	Gln	Asn	Asn	Ala
	34	HRB-I3	KT074309	Dec-2014	HRB	NA	F	3M	Yes		New2a	Ala	Ile	Gln	Asn	Asn	Ala
	35	HRB-I4	KT074310	Sep-2014	HRB	SN	M	3.5M	NA	—	New2a	Ala	Ile	Gln	Asn	Asn	Ala
	36	HRB-I8	KT074312	Feb-2015	HRB	MI	M	3M	No	—	New2a	Ala	Ile	Gln	Asn	Asn	Ala
	37	HRB-J1	KT074313	Sep-2014	HRB	NA	M	3M	Yes	—	New2a	Ala	Ile	Gln	Asn	Asn	Ala
	38	HRB-K1	KT074316	Mar-2015	HRB	NA	M	3.5M	Yes	—	New2a	Ala	Ile	Gln	Asn	Asn	Ala
	39	HRB-K3	KT074317	Jan-2015	HRB	NA	F	3M	Yes	CaKV (KT210425)	New2a	Ala	Ile	Gln	Ser	Asn	Ala
	40	HRB-G0	KT074304	Dec-2014	HRB	PK	M	4M	Yes	—	New2a	Ala	Ile	Gln	Ser	Asn	Ala
	41	HRB-F8	KT074303	Nov-2014	HRB	PD	M	3M	No	CCoV (KT192691), CaKV (KT210418)	New2a	Ala	Ile	Gln	Ser	Asn	Ala
	42	MDJ-7	KT074259	Nov-2014	MDJ	NA	M	6M	Yes	—	New2a	Ala	Ile	Gln	Asn	Asn	Thr
	43	MDJ-9	KT074260	Dec-2014	MDJ	RC	M	3M	No	—	New2a	Ala	Ile	Gln	Asn	Asn	Thr
	44	MDJ-15	KT074261	Nov-2014	MDJ	RC	F	1.5M	Yes	CBoV (KR998482)	New2a	Ala	Ile	Gln	Asn	Asn	Thr
	45	MDJ-18	KT074262	Mar-2015	MDJ	MX	M	3M	Yes	—	New2a	Ala	Ile	Gln	Asn	Asn	Thr
	46	MDJ-20	KT074263	Mar-2015	MDJ	NA	F	3M	Yes	—	New2a	Ala	Ile	Gln	Asn	Asn	Thr
	47	MDJ-21	KT074264	Dec-2014	MDJ	RC	M	3M	No	CBoV (KR998484)	New2a	Ala	Ile	Gln	Asn	Asn	Thr
	48	MDJ-27	KT074266	Apr-2015	MDJ	NA	F	NA	NA	CBoV (KR998487), CaKV (KT210392)	New2a	Ala	Ile	Gln	Asn	Asn	Thr
	49	MDJ-33	KT074270	Jan-2015	MDJ	MX	NA	3M	No	—	New2a	Ala	Ile	Gln	Asn	Asn	Thr
	50	MDJ-37	KT074271	Apr-2015	MDJ	NA	M	NA	Yes	—	New2a	Ala	Ile	Gln	Asn	Asn	Thr
	51	DQ-beta7	KT074275	Feb-2015	DQ	NA	M	1M	No	CBoV (KR998491)	New2a	Ala	Ile	Gln	Asn	Asn	Thr
	52	HRB-A2	KT074277	Sep-2014	HRB	CH	M	4M	No	—	New2a	Ala	Ile	Gln	Asn	Asn	Thr
	53	HRB-B8	KT074282	Oct-2014	HRB	GM	NA	NA	NA	—	New2a	Ala	Ile	Gln	Asn	Asn	Thr
	54	HRB-C7	KT074288	Oct-2014	HRB	NA	M	2M	NA	CBoV (KR998493)	New2a	Ala	Ile	Gln	Asn	Asn	Thr
	55	HRB-a1	KT074276	Sep-2014	HRB	NA	F	3.5M	Yes	CCoV (KT192656), CBoV (KR998488), CaKV (KT210394)	New2a	Ala	Ile	Gln	Asn	Asn	Thr
	56	HRB-ee7	KT074292	Dec-2014	HRB	NA	M	3.5M	Yes	—	New2a	Ala	Ile	Gln	Asn	Asn	Thr
	57	HRB-D2	KT074293	Oct-2014	HRB	PD	F	2M	No	—	New2a	Ala	Ile	Gln	Asn	Asn	Thr
	58	HRB-G6	KT074306	Dec-2014	HRB	GM	F	3.5M	Yes	—	New2a	Ala	Ile	Gln	Asn	Asn	Thr
	59	HRB-I7	KT074311	Nov-2014	HRB	NA	F	NA	Yes	—	New2a	Ala	Ile	Gln	Asn	Asn	Thr
	60	HRB-J4	KT074314	Oct-2014	HRB	NA	M	4M	No	—	New2a	Ala	Ile	Gln	Asn	Asn	Thr
	61	HRB-J7	KT074315	Nov-2014	HRB	NA	F	3.5M	Yes	—	New2a	Ala	Ile	Gln	Asn	Asn	Thr
	62	DQ-beta0	KT074336	Dec-2014	DQ	PM	M	1.5M	No	CCoV (KT192673)	New2b	Ala	Ile	Gln	Asn	Asp	Ala
	63	DQ-beta4	KT074337	Dec-2014	DQ	SN	F	5M	Yes	—	New2b	Ala	Ile	Gln	Asn	Asp	Ala
	64	DQ-beta9	KT074338	Mar-2015	DQ	CO	M	NA	Yes	—	New2b	Ala	Ile	Gln	Asn	Asp	Ala
	65	HRB-A4	KT074319	Sep-2014	HRB	NA	F	3M	NA	CCoV (KT192652), CaKV (KT210396)	New2b	Ala	Ile	Gln	Asn	Asp	Ala
	66	HRB-A9	KT074320	Sep-2014	HRB	PM	NA	3M	NA	—	New2b	Ala	Ile	Gln	Asn	Asp	Ala
	67	HRB-B6	KT074321	Oct-2014	HRB	CH	F	2M	No	—	New2b	Ala	Ile	Gln	Asn	Asp	Ala
	68	HRB-aa8	KT074322	Oct-2014	HRB	BF	M	4M	NA	—	New2b	Ala	Ile	Gln	Asn	Asp	Ala
	69	HRB-b2	KT074323	Oct-2014	HRB	GM	NA	3M	NA	CBoV (KR998490)	New2b	Ala	Ile	Gln	Asn	Asp	Ala
	70	HRB-d1	KT074324	Nov-2014	HRB	LR	F	7M	Yes	—	New2b	Ala	Ile	Gln	Asn	Asp	Ala
	71	HRB-d3	KT074325	Oct-2014	HRB	RC	NA	NA	NA	—	New2b	Ala	Ile	Gln	Asn	Asp	Ala
	72	HRB-d5	KT074326	Sep-2014	HRB	NA	M	3M	No	—	New2b	Ala	Ile	Gln	Asn	Asp	Ala
	73	HRB-E7	KT074327	Nov-2014	HRB	SM	F	3M	Yes	—	New2b	Ala	Ile	Gln	Asn	Asp	Ala
	74	HRB-F5	KT074328	Nov-2014	HRB	YT	F	4M	Yes	CCoV (KT192690)	New2b	Ala	Ile	Gln	Asn	Asp	Ala
	75	HRB-F6	KT074329	Nov-2014	HRB	NA	M	3M	No	—	New2b	Ala	Ile	Gln	Asn	Asp	Ala
	76	HRB-F7	KT074330	Nov-2014	HRB	MI	F	3M	Yes	—	New2b	Ala	Ile	Gln	Asn	Asp	Ala
	77	HRB-H3	KT074331	Jan-2015	HRB	SM	M	3M	No	CCoV (KT192693)	New2b	Ala	Ile	Gln	Asn	Asp	Ala
	78	HRB-H4	KT074332	Dec-2014	HRB	NA	F	3.5M	No	—	New2b	Ala	Ile	Gln	Asn	Asp	Ala
	79	HRB-H8	KT074333	Dec-2014	HRB	BF	M	3.5M	No		New2b	Ala	Ile	Gln	Asn	Asp	Ala
	80	HRB-I5	KT074334	Oct-2014	HRB	BD	M	3.5M	Yes	—	New2b	Ala	Ile	Gln	Asn	Asp	Ala
	81	HRB-J8	KT074335	Dec-2014	HRB	SM	NA	3.5M	Yes	—	New2b	Ala	Ile	Gln	Asn	Asp	Ala
	82	MDJ-4	KT074348	Apr-2015	MDJ	NA	M	4M	No	—	2c	Ala	Ile	Arg	Asn	Glu	Thr
	83	MDJ-6	KT074349	Dec-2014	MDJ	NA	F	NA	Yes	—	2c	Ala	Ile	Arg	Asn	Glu	Thr
	84	MDJ-11	KT074350	Oct-2014	MDJ	PD	F	4M	No	CCoV (KT192644)	2c	Ala	Ile	Arg	Asn	Glu	Thr
	85	MDJ-24	KT074351	Nov-2014	MDJ	RC	F	1.5M	Yes	—	2c	Ala	Ile	Arg	Asn	Glu	Thr
	86	MDJ-39	KT074352	Mar-2015	MDJ	NA	F	3M	No	—	2c	Ala	Ile	Arg	Asn	Glu	Thr
	87	HRB-A6	KT074339	Sep-2014	HRB	NA	F	3M	Yes	CCoV (KT192653)	2c	Ala	Ile	Arg	Asn	Glu	Thr
	88	HRB-aa9	KT074340	Oct-2014	HRB	CH	M	3.5M	No		2c	Ala	Ile	Arg	Asn	Glu	Thr
	89	HRB-b3	KT074341	Oct-2014	HRB	PM	NA	2M	No	CCoV (KT192670), CaKV (KT210409)	2c	Ala	Ile	Arg	Asn	Glu	Thr
	90	HRB-E5	KT074342	Nov-2014	HRB	NA	M	3M	Yes	—	2c	Ala	Ile	Arg	Asn	Glu	Thr
	91	HRB-e6	KT074343	Mar-2015	HRB	PD	F	3.5M	No	—	2c	Ala	Ile	Arg	Asn	Glu	Thr
	92	HRB-E9	KT074344	Nov-2014	HRB	CH	NA	NA	Yes	—	2c	Ala	Ile	Arg	Asn	Glu	Thr
	93	HRB-F9	KT074345	Feb-2015	HRB	NA	M	NA	NA	—	2c	Ala	Ile	Arg	Asn	Glu	Thr
	94	HRB-G8	KT074346	Nov-2014	HRB	PM	NA	4M	Yes	CaKV (KT210421)	2c	Ala	Ile	Arg	Asn	Glu	Thr
	95	HRB-J6	KT074347	Nov-2014	HRB	PD	NA	NA	Yes	—	2c	Ala	Ile	Arg	Asn	Glu	Thr

***Note***. For breed, GM = Golden Malinois, LR = Labrador Retriever, RC = Rough Collie, CO = Caucasian Owtcharka, TM = Tibetan Mastiff, PD = Poodle, CH = Chihuahua, and JS = Japanese Spitz; For gender, F = female, and M = male; for age, M = month; for location, MDJ = Mudanjiang, HRB = Harbin, DQ = Daqing.

**Table 3:** Further analysis of the CPV-2 positive samples.

				Harbin	Daqing	Mudanjiang	Total
**Numbers of sample**				141	20	40	201
**Positive rate for CPV-2**				48.94% (69/141)	30% (6/20)	50% (20/40)	47.26% (95/201)
**Genotyping of CPV-2**		New2a		62.32% (43/69)	50% (3/6)	75% (15/20)	64.21% (61/95)
		New2b		24.64% (17/69)	50% (3/6)	—	21.05% (20/95)
		2c		13.04% (9/69)	—	25% (5/20)	14.74% (14/95)
**Substitution of amino acid residues in VP2 protein of CPV-2**		^297^Ser/Ala	Ser	—	—	—	—
			Ala	100% (69/69)	100% (6/6)	100% (20/20)	100% (95/95)
		^324^Tyr/Ile	Tyr	—	—	—	—
			Ile	100% (69/69)	100% (6/6)	100% (20/20)	100% (95/95)
		^370^Gln/Arg	Gln	86.96% (60/69)	100% (6/6)	75% (15/20)	85.26% (81/95)
			Arg	13.04% (9/69)	—	25% (5/20)	14.74% (14/95)
		^419^Asn/Ser	Asn	95.65% (66/69)	100% (6/6)	100% (20/20)	96.84% (92/95)
			Ser	4.35% (3/69)	—	—	3.16% (3/95)
		^440^Thr/Ala	Thr	27.54% (19/69)	16.67% (1/6)	70% (14/20)	35.79% (34/95)
			Ala	72.46% (50/69)	83.33% (5/6)	30% (6/20)	64.21% (61/95)
**Vaccined**		Yes		44.93% (31/69)	50% (3/6)	60% (12/20)	48.42% (46/95)
		No		30.43% (21/69)	50% (3/6)	35% (7/20)	32.63% (31/95)
**Collection data**		Jan. to Mar.		11.59% (8/69)	33.33% (2/6)	25% (5/20)	15.78% (15/95)
		Apr. to Jun.		1.45% (1/69)	16.67% (1/6)	25% (5/20)	7.37% (7/95)
		Jul. to Sep.		15.94% (11/69)	—	—	11.58% (11/95)
		Oct. to Dec.		71.01% (49/69)	50% (3/6)	50% (10/20)	65.26% (62/95)
**Age**		0< Age ≤2M		8.70% (6/69)	33.33% (2/6)	15% (3/20)	11.58% (11/95)
		2M< Age ≤4M		73.91% (51/69)	33.33% (2/6)	45% (9/20)	65.26% (62/95)
		4M< Age ≤6M		1.45% (1/69)	16.67% (1/6)	15% (3/20)	5.26% (5/95)
		>6M		1.45% (1/69)	—	—	1.05% (1/95)
**Other enteric viruses in the CPV positive samples**		CCoV		24.64% (17/69)	33.33% (2/6)	10% (2/20)	22.11% (21/95)
		CaKV		18.84% (13/69)	—	5% (1/20)	14.74% (14/95)
		CBoV		4.35% (3/69)	16.67% (1/6)	15% (3/20)	7.37% (7/95)
		CCoV+CaKV		13.04% (9/69)	—	5% (1/20)	10.53% (10/95)
		CBoV+CaKV		1.45% (1/69)	—	5% (1/20)	2.11% (2/95)
		CCoV+CBoV+CaKV		1.45% (1/69)	—	—	1.05% (1/95)
**Identity**	Nucleotides	New2a		99.2%–100%	99.2%–100%	99.2%–100%	99.0%–100%
		New2b		99.6%–100%	100%	—	99.8%–100%
		2c		100%	—	100%	100%
		New2a+ New2b+2c		98.8%–100%	99.0%–100%	98.6%–100%	98.8%–100%
	Amino acids	New2a		98.8%–100%	99.4%–100%	99.4%–100%	98.8%–100%
		New2b		100%	100%	—	100%
		2c		100%	—	100%	100%
		New2a+ New2b+2c		97.6%–100%	98.8%–100%	98.2%–100%	97.6%–100%

Sequence comparisons revealed 99.0%–100%, 99.8%–100%, and 100% nucleotide identities within the new2a, new2b, and 2c strains, respectively, and 98.8%–100% nucleotide identities between the new2a, new2b, and 2c types, respectively. At the amino acid level, 98.8%–100%, 100%, 100%, 97.6%–100% identities were revealed within the new2a, new2b, and 2c strains, and between the new2a, new2b, and 2c types, respectively. A total of five substitutions of amino acid residues occurred in all 95 CPV-2-positive samples based on an analysis of partial VP2 sequences. Residue ^324^Ile was present in all 95 CPV-2-positive samples when compared with the reference strains, and residue ^370^Arg was specific for type CPV-2c when compared with CPV-2c reference strains. Only three new CPV-2a strains, HRB-K3, HRB-G0, and HRB-F8, showed amino acids substitutions (Asn→Ser) at position 419. At position 440, all new2b strains and most of the new2a strains identified in our study showed amino acids substitutions (Thr→Ala) when compared with the reference strains.

For the CPV-2-positive animals, 65.26% (62/95) were aged from 2–4 months, 48.42% (46/95) had a vaccination history, and 32.63% (31/95) had no vaccination history, while 65.26% (62/95) were collected from October to December. Coinfections with CCoV, CaKV, and CBoV were found in the 95 CPV-2-positive samples, of which 22.11% (21/95) were positive for CCoV, 14.74% (14/95) were positive for CaKV, 7.37% (7/95) were positive for CBoV, 10.53% (10/95) were positive for CCoV and CaKV, 2.11% (2/95) were positive for CBoV and CaKV, and 1.05% (1/95) were positive for CCoV, CBoV, and CaKV.

### Phylogenetic analysis

**Fig 1:**
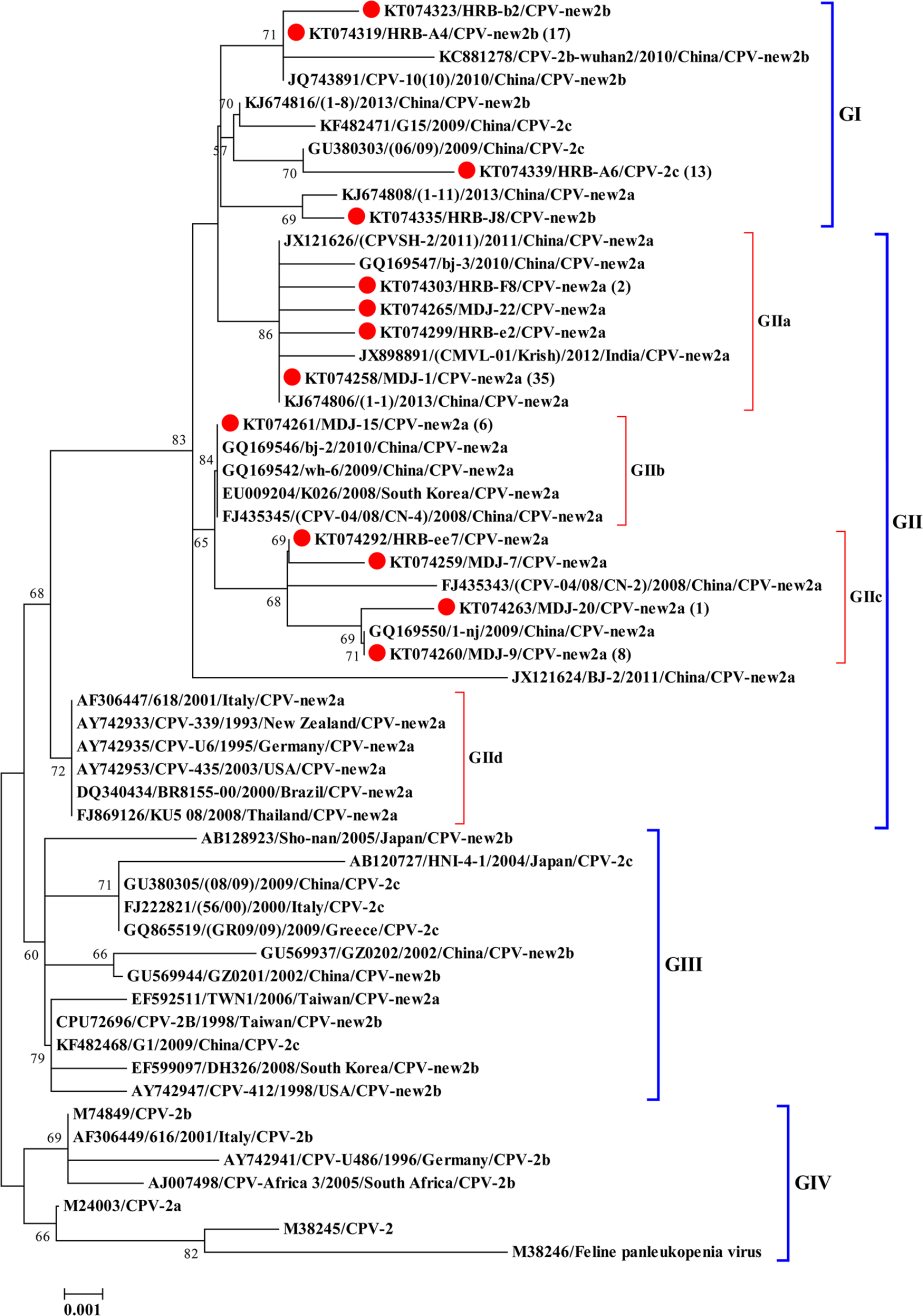
Phylogenetic analysis of CPV-2 strains on the basis of the partial VP2 gene (507 bp). The red spot diagram represents CPV-2 strains identified in this study. The values in parentheses indicate the number of strains with identical sequences to that included in the tree. (1): KT074275 from Daqing; (2): KT074317 and KT074304 from Harbin; (6): KT074266, KT074270 from Mudanjiang; KT074276, KT074288, KT074293, and KT074306 from Harbin; (8): KT074262, KT074264, and KT074271 from Mudanjiang; KT074277, KT074282, KT074311, KT074314, and KT074315 from Harbin. (13): KT074340-KT074347 from Harbin; KT074348-KT074352 from Mudanjiang. (17): KT074320-KT074322 and KT074324-KT074334 from Harbin; KT074336-KT074338 from Daqing; (35): KT074267-KT074269 and KT074272 from Mudanjiang; KT074273 and KT074274 from Daqing; KT074278, KT074279, KT074280-KT074287, KT074289- KT074291, KT074294- KT074298, KT074300- KT074302, KT074305, KT074307- KT074310, KT074312, KT074313, KT074316, and KT074318 from Harbin.

To analyze the genetic diversity of the new2a, new2b, and 2c strains identified in northeast China, partial VP2 gene sequences of CPV-2, CPV-2a, CPV-2b, CPV-2c, new CPV-2a, and new CPV-2b strains from different geographical locations within China and the rest of the world were used to construct a phylogenetic tree. Nucleotide sequences of the partial VP2 used for construction of phylogenetic tree was shown in Supporting Information (S2 Fig). The generated phylogenetic tree was composed of four groups: GI, GII, GIII, and GIV. The 95 CPV-2 strains from northeast China were divided into two different groups: GI and GII (Fig 1). All 14 CPV-2c strains and 20 new CPV-2b strains identified in our study were divided into the GI group. Meanwhile, the GI group included the new2a, new2b and 2c reference strains from China. The GII group, consisting of four subgroups, GIIa, GIIb, GIIc, and GIId, was specific for the new CPV-2a strains, except for one new CPV-2b strain (HRB-J8). The new CPV-2a strains identified in northeast China were divided into the three subgroups GIIa, GIIb, and GIIc, and the new CPV-2a reference strains from other countries were divided into the subgroup GIId. The GIV group consisted of different genotypes of CPV-2 reference strains from China and other countries.

### Recombination analysis

**Fig 2:**
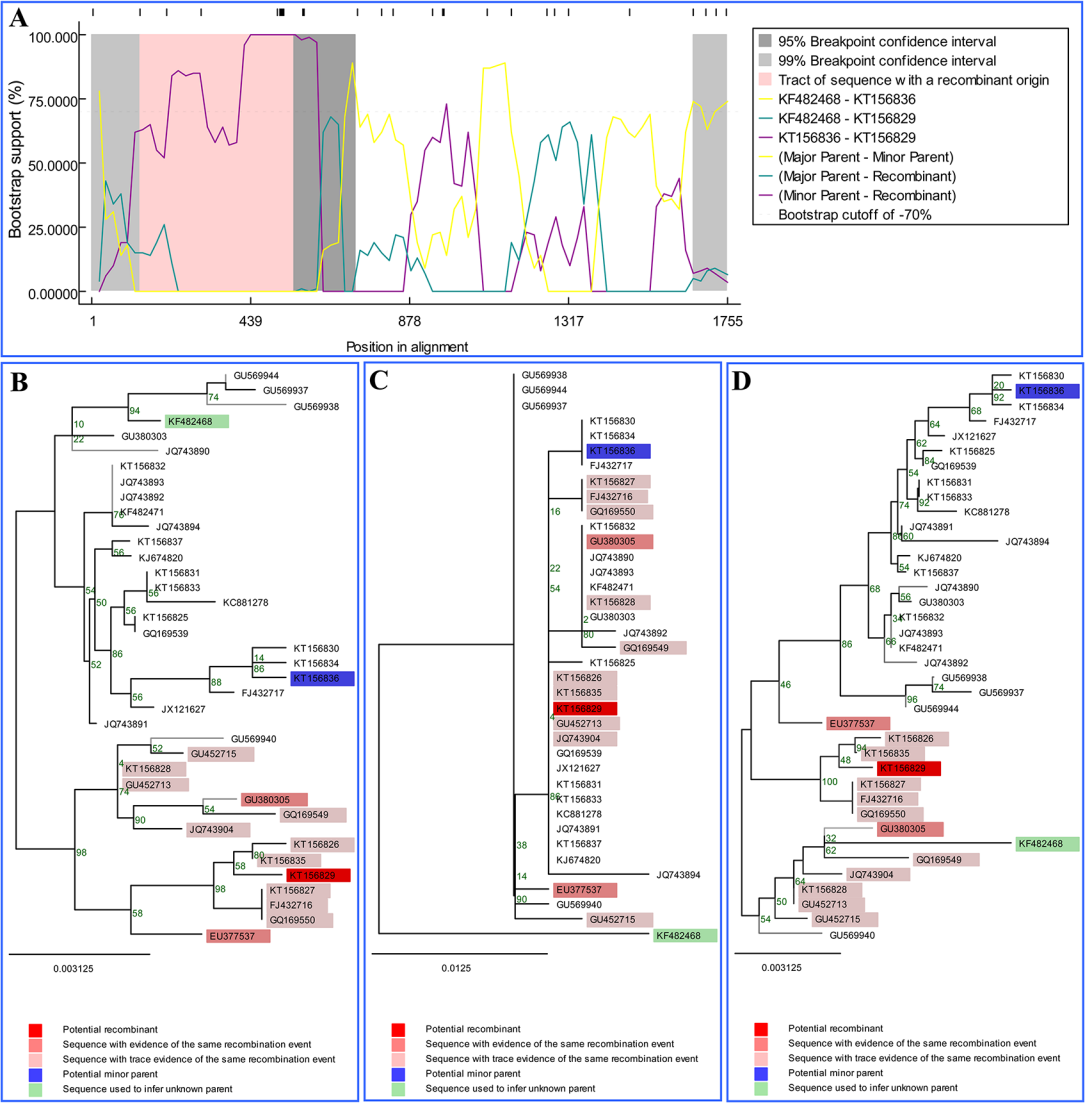
Identification of the recombination event between the minor parent strain HRB-F8 (KT156836) (blue) and the major parent strain G1 (KF482468) (green), which led to the recombinant MDJ-20 strain (KT156829) (red). (A) Bootscan evidence for the recombination origin on the basis of pairwise distance, modelled with a window size 200, step size 20 and 100 bootstrap replicates. (B, C, and D) Fast neighbour-joining (NJ) tree (1000 replicates, Kimura two-parameter distance) constructed using the regions derived from minor parent strain (1–133, 558–1755) (B), the recombination region (134–557) (C), and non-recombinant region (D). *Note*. The potential recombination event was detected in Maxchi (*P* = 3.74E-02) and SiSscan (*P* = 1.42E-04) methods.

**Fig 3:**
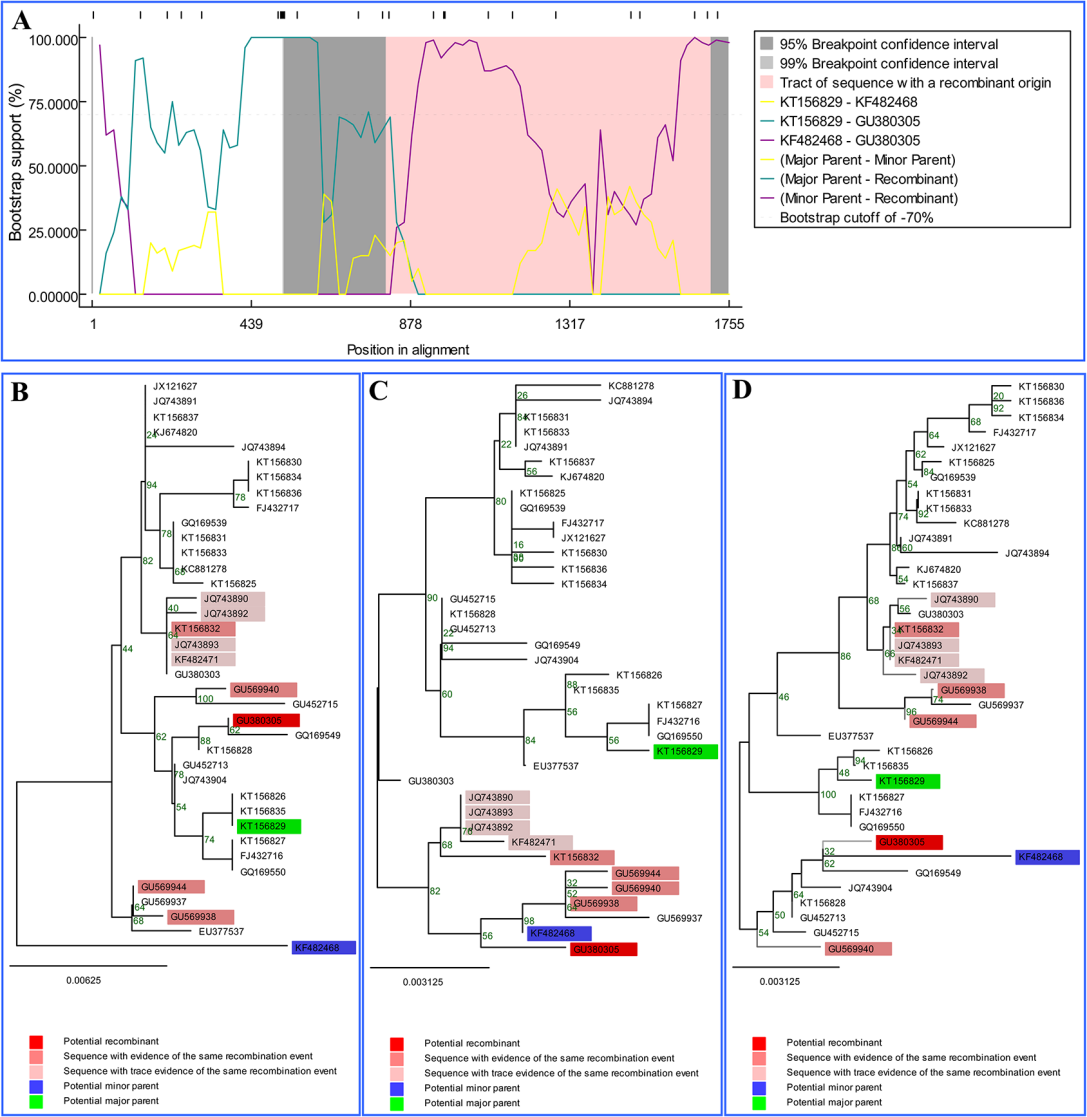
Identification of the recombination event between the major parent strain MDJ-20 (KT156829) (green) and the minor parent strain G1 (KF482468) (blue), which led to the recombinant 08/09 strain (GU380305) (red). (A) Bootscan evidence for the recombination origin on the basis of pairwise distance, modelled with a window size 200, step size 20 and 100 bootstrap replicates. (B, C, and D) Fast neighbour-joining (NJ) tree (1000 replicates, Kimura two-parameter distance) constructed using the regions derived from minor parent strain (1–809, 1708–1755) (B), the recombination region (810–1707) (C), and non-recombinant region (D). *Note*. The potential recombination event was detected in Maxchi (*P* = 8.51E-04), SiSscan (*P* = 1.05E-02), and Chimaera (*P* = 7.25E-03) methods.

The recombination analysis based on the VP2 gene revealed that two potential recombination events occurred between the selected 13 strains in this study and the 25 reference strains from China, but no potential recombination events were found within the selected 13 strains in this study. Nucleotide sequences of the entire VP2 of CPV-2 strains identified in our study were shown in Supporting Information (S3 Fig), and the breakpoint positions of recombination events were shown in Supporting Information (S4 Fig). Fig 2 shows the recombination event that occurred between the HRB-F8 strain (accession no. KT156836) and a G1 strain (accession no. KF482468), which led to the recombinant MDJ-20 strain (accession no. KT156829). The bootscan plot of this recombination event is shown in Fig 2A, which used HRB-F8 and G1 as minor parent strain, and major parent strain, respectively. The recombination event was confirmed by fast neighbor-joining trees that were constructed using the regions derived from minor parent strain (1–133 and 558–1755) (Fig 2B), the recombinant region (134–557) (Fig 2C), and the non-recombinant region (Fig 2D). Fig 3 shows the recombination event that occurred between MDJ-20 strain (KT156829) and a G1 strain (KF482468), which led to the recombinant 08/09 strain (GU380305). The bootscan plot of this recombination event is shown in Fig 3A, which used MDJ-20 and G1 as major parent strain, and minor parent strain, respectively. The recombination event was confirmed by fast neighbor-joining trees that were constructed using the regions derived from minor parent strain (1–809 and 1708–1755) (Fig 3B), the recombinant region (810–1707) (Fig 3C), and the non-recombinant region (Fig 3D). The two recombinant strains MDJ-20 and 80/09 were divided into different clusters in the recombination region-based phylogenetic trees when compared with the non-recombination region-based phylogenetic trees.

## Discussion

It has been reported that a high proportion (29.2%–84%) of fecal samples from diarrhea-affected dogs tested positive for CPV-2 [11, 29]. In our study, 201 samples were collected from diarrhea-affected dogs in three districts in Heilongjiang province in northeast China, and 95 CPV-2-positive samples were identified, accounting for 47.26% of all samples. The results were in line with recent reports in China [15, 30]. The CPV-2-positive rate differed among the three districts (Harbin, 48.94%; Daqing, 30%; Mudanjiang, 50%). In our study, mixed infections of CCoV (22.11%), CaKV (14.74%), and CBoV (7.37%) were found in the CPV-2-positive samples. A coinfection of CCoV, CBoV, and CaKV with CPV-2 was also found in one sample. Cavalli et al. (2014) reported that mixed infections of CPV-2 and CCoV reached 49.12% in diarrheic dogs in Albania [31]. CaKV and CBoV had been reported to be associated with severe enteritis in a litter of puppies [28, 32]. The high prevalence of co-infections of CPV-2 with three enteric viruses may be associated with viral diarrhea in dogs in northeast China.

In our study, CPV-2-positive rates showed clear differences among seasons and ages. 65.26% of CPV-2-positive samples were collected from October to December. The high prevalence of CPV-2 between October and December may be indicative of a seasonal variation in northeast China. Additionally, a high CPV-2-positive rate was found in animals aged from 2–4 months, which is similar to the report described by Cavalli et al. (2014) [31]. At present, most dogs in China are vaccinated for CPV-2. However, in the CPV-2-positive samples identified in this study, 48.42% came from dogs with a vaccination history. This data demonstrated that the CPV-2 vaccines used in northeast China may fail to generate protective antibody titers against heterogeneous CPV antigenic types.

The Ser297Ala mutation may have had a remarkable influence on the process of host adaptation [33]. This mutation was used as a marker of the new CPV-2a/2b variant. This study indicated that the new CPV-2a (Ser297Ala) variant, accounting for 64.21% of CPV-2 infections, was the predominating strain circulating in the three districts of Heilongjiang province in northeast China. The new CPV-2b (Ser297Ala) variant, accounting for 21.05% of CPV-2 infections, was the second most predominant strain circulating in the Harbin and Daqing districts of Heilongjiang province. In our study, the prevalence of the new CPV-2a is in agreement with most of the reports that were recently conducted in China [8, 15, 30, 34, 35], African countries [36], other Asian countries, including Korea, Thailand, Taiwan, and Turkey, and Australia [6, 37–39]. However, the prevalence of the new CPV-2b variant reported here in CPV-2-positive samples was higher than those in other reports in China. Interestingly, the presence of the rare CPV-2c, accounting for 14.74% of the CPV-2-positive samples, was confirmed in the Harbin and Mudanjiang of Heilongjiang province. The co-circulation of the CPV-2c, new CPV-2a, and new CPV-2b has previously not been reported in China. It has been reported that the CPV-2c variant is widely distributed and co-exists with other CPV types in Europe and North and South America [40–45]. Compared with an earlier report that detected the CPV-2, new CPV-2a, and CPV-2b variants in China [46], the emergence of the CPV-2c in northeast China suggests that CPV-2 strains circulating in China exhibit genetic variations. Additionally, our results are similar to those reported for the genotypes of CPV-2 strains circulating in India, where the new CPV-2a and new CPV-2b were the predominant strains [14], and the co-circulation of the CPV-2c was also found [47].

In the VP2 protein of CPV-2, the Tyr324Ile mutation is adjacent to residue 323, which affects binding to canine transferrin receptors, resulting in changes in the host range of canine parvovirus [13]. Presumably, this mutation may result in stronger receptor binding. In our study, all 95 identified CPV-2 strains, including the new2a, new2b, and 2c types, contained the unique Tyr324Ile mutation. The Tyr324Ile mutation in CPV-2a strains has been reported in China [8, 15, 30, 46], South Korea [38], Thailand [48], Uruguay [49], Japan [50], Taiwan [6], and India [51]. These data support the view that the Try324Ile substitution in CPV-2 strains may be a common amino acid alteration in Asia countries, especially in China. This conclusion needs to be validated by an extensive epidemiological survey in a future study. In addition, further studies should be conducted to understand the relationship between the Try324Ile substitution and the severity of clinical symptoms.

In the VP2 protein of CPV-2, the amino acid residue at position 440 is located in the GH loop. It has been reported that the high levels of the Thr440Ala substitution in VP2 are associated with the evolution of antigenic variants in circulating parvovirus types [52, 53]. In our study, the Thr440Ala substitution was found in 64.21% of the 95 CPV-2-positive samples, of which 100% of the new2b strains had the Thr440Ala substitution, 67.22% of the new2a strains exhibited the Thr440Ala substitution, while the type 2c strains did not have the Thr440Ala substitution. At present, the Thr440Ala substitution has been frequently reported in China and other countries [8, 14, 15, 36]. Therefore, we suggest further studies to better understand the relationship between this mutation and the severity of clinical symptoms.

In this study, both new CPV-2b strains and CPV-2c strains exhibited 100% amino acid identity on the basis of the deduced sequence of the partial VP2 protein among different districts. These data demonstrated that type new2b and 2c strains of CPV-2 circulating in northeast China exhibited genetic stability. However, compared with CPV-2 reference strains, the type new2b strains showed a unique Thr440Ala substitution (100%), and the type 2c strains exhibited a unique Gln370Arg substitution (100%). The amino acid substitutions resulted in phylogenetic changes in the new2b and 2c strains identified in our study. The new2b and 2c strains (except one strain) differed genetically from the reference strains of other countries, forming one group (GI) with Chinese reference strains of the new2b and 2c types. In our study, the close relationship between the new 2b and 2c types suggests that these two types share a similar evolutionary history in China. For the identified type 2c strains, the unique Gln370Arg substitution, which was previously not described, was highlighted as evidence of a potential CPV-2c variant or new CPV-2c. Further studies regarding potential variant CPV-2c strains should be conducted to understand the relationship between the Gln370Arg substitution and viral pathogenicity.

The phylogenetic analysis revealed that the type new2a strains identified in our study exhibited high variability, forming three subgroups. Of the 61 new2a strains, a total of two amino acid substitution sites were revealed, Asn419Ser and Thr440Ala. The two amino acid substitutions, as well as some non-synonymous substitutions, accounted for the genetic diversity of the new2a strains in the phylogenetic tree. In our study, the Asn419Ser substitution was detected in only three of the 61 new2a strains. We suggest that the Asn419Ser substitution, first described here, should be the focus of further epidemiological investigations of CPV-2. Accumulating reports have indicated that the new CPV-2a strains, accounting for high positive rates among CPV-2 types, are the predominating strains circulating in China. The high variability of the new CPV-2a strains in our study may be attributed to long-term interactions between the new CPV-2a strains and their hosts. It is suggested that vaccinations against CPV-2 should be updated on the basis of these new CPV-2a strains.

Although point mutations are considered the main mechanism for generating genomic diversity in CPV, co-infection and recombination have also been explored as variability-inducing mechanisms [54]. Pérez et al. (2014) revealed that a recombinant strain arose from inter-genotypic recombination between CPV-2c and CPV-2a strains within the VP1/VP2 gene boundary [55]. In our study, two inter-genotypic recombination events between the 13 CPV-2 strains in this study and the Chinese reference strains were identified in the VP2 gene. The two recombination events occurred between type new2a and 2c strains, resulting in two potential recombinant type new2a strains. Our finding is in line with the occurrence of a possible recombination event in the CPV-2 strain 364-rec, as described by Pérez et al. (2014) [55]. Although the evidence of naturally occurring recombination events was not obtained in our study, possible recombination events among Chinese CPV-2 strains suggest that co-infections with CPV-2 strains with different genotypes, especially types 2a and 2c, as well as potential recombinant strains of CPV-2, should be monitored in China by extensive molecular epidemiology investigations in the future.

Since 1983, the epidemiological information of CPV-2 strains circulating in China has been frequently reported [8, 15, 30, 34, 35, 46]. Accumulating reports indicated that circulation of CPV-2, CPV-2a, CPV-2b, new CPV-2a, and new CPV-2b strains was identified in different districts of China, of which new CPV-2a was predominant strains in China, followed by new CPV-2b. Compared with the previous epidemiological investigation of CPV-2 in China, the detailed information of samples used in our study, including collection date, location, animal breed, animal gender, animal age, animal immunization, and coinfection with other enteric viruses, resulted in the generation of more precise and valuable epidemiological data which not only enriches basic epidemiological data of Chinese CPV-2 strains, but also increases the understanding of epidemic characteristics of CPV-2 strains circulating in northeast China. In addition, our findings revealed novel epidemiological information of CPV-2 in China, involving in the co-circulation of new CPV-2a with high variation, new CPV-2b, and rare CPV-2c in Heilongjiang province of northeast China, the unique amino acids substitutions including Gln370Arg substitution in identified CPV-2c strains, and Thr440Ala substitution in identified new CPV-2b strains, and the potential recombination events which could happen among type new2a and 2c CPV-2 field strains circulating in China.

## Conclusions

Taken together, in the current study, the resulting data increase our understanding of the genetic evolution of CVP-2 strains in China, and they also provide valuable information for further studies of CPV-2 in other countries. However, some speculations resulting from our data still need to be validated by further and extensive epidemiological investigation in the future.

## Supporting Information

S1 Fig Nucleotide sequences of the partial VP2 gene of CPV-2 strains identified in our study. (TXT)

S2 Fig Nucleotide sequences of the partial VP2 gene were used for construction of phylogenetic tree. (TXT)

S3 Fig Nucleotide sequences of the entire VP2 gene of CPV-2 strains identified in our study were used for analysis of recombination events. (TXT)

S4 Fig The breakpoint positions of recombination events in our study. (XLS)
